# What influences informal caregivers' risk perceptions and responses to home care safety of older adults with disabilities: A qualitative study

**DOI:** 10.3389/fpubh.2022.901457

**Published:** 2022-08-24

**Authors:** Songmei Cao, Huanhuan Huang, Suping Bo, Man Feng, Yiqing Liang, Yuqing Liu, Qinghua Zhao

**Affiliations:** ^1^School of Nursing, Chongqing Medical University, Chongqing, China; ^2^Department of Nursing, First Affiliated Hospital of Chongqing Medical University, Chongqing, China; ^3^Department of Nursing, Affiliated Hospital of Jiangsu University, Zhenjiang, China; ^4^Global Health Research Center, Duke Kunshan University, Kunshan, China

**Keywords:** informal caregiver, home care, safety, risk perception, older adult

## Abstract

**Objective:**

This study aimed to explore the factors that influence risk perceptions and responses by informal caregivers of older adults with disabilities.

**Methods:**

A descriptive qualitative study was performed, and the socio-ecological framework was applied to interpret the complex influences on individual risk perceptions and responses. Semistructured interviews were conducted with 16 informal caregivers of older adults with disabilities. The interviews were transcribed verbatim and analyzed using content analysis.

**Results:**

The four levels of the socio-ecological framework successfully allowed for the analysis of influences on the risk perceptions and responses of informal caregivers as follows: at the individual level: previous experiences, personality characteristics, health literacy, and care burden; at the familial level: economic status, emotional connection, informational and decisional support; at the community level: health service accessibility and neighbor communication; and at the social level: responsibility-driven culture, media advocacy, and aging policies.

**Conclusions:**

The establishment of risk perceptions and coping behaviors by informal caregivers was affected by many factors. Using the framework to interpret our findings provided insight into the influence of these varying factors. Comprehensive, realistic, and achievable strategies are needed for improving the risk perceptions of informal caregivers in home care by addressing personal, familial, and social environmental factors.

## Introduction

Population aging constitutes a significant public health challenge globally. According to the World Population Prospects 2019 of the United Nations, the global population of people aged 65 years and over is expected to more than double by 2050 (1). With the aging of populations worldwide, the corresponding increase in the number of older adults with disabilities is becoming a significant concern (2). China has the largest elderly population in the world. There were approximately 40.63 million disabled or semidisabled elderly individuals in China (3). Ninety percent of elderly individuals with a disability choose to live at home (4). Disability is commonly defined as experiencing difficulty in performing activities that are essential for independent living (5, 6). Older adults with disabilities are at a higher risk of safety-related events than the general population because of their poor health status. Unique family situations, the need for medical devices, and the availability of care resources complicate home care safety for older adults. Most community-residing older adults who need assistance receive care exclusively from informal sources (7). Caregivers are generally family members, friends, or acquaintances, and they are often referred to as informal caregivers (8). Informal caregivers' abilities to identify and avoid risks are very important to ensuring home safety for older adults. While safety issues in the home are multifaceted, in this article, the term “safety” refers to health-related safety, which is defined as minimizing the probability of preventable, unintended harm to community-dwelling individuals (9). Previous studies reported that common adverse health events in home care include falls, pressure sores, adverse drug events, healthcare-associated infections, unplanned extubations, and so on (10–14).

Interpretations and other subjective judgments about risks are known as risk perceptions (15). In health-related areas, risk perception generally refers to subjective judgments about the likelihood of negative occurrences such as injury, illness, disease, and death (16). Risk perception is a complex process that includes the ways people think, feel, and behave in response to risk (17). Risk perception has an important impact on many aspects of behavior because it identifies which hazards people care about and how they deal with them. A meta-analysis of experimental research showed that the higher the risk of an unfavorable health outcome is perceived to be, the more likely people are to engage in healthier behaviors (18). The influence of risk perception on health-related decisions and behaviors also occurs when people make health decisions on behalf of another person who depends on them (19). It is therefore important to study the risk perceptions and responses of caregivers regarding the care of older adults with disabilities.

Risk perception has been studied extensively. In recent years, there has been a notable increase in the number of studies in public health on topics such as risk perceptions of a disease outbreak or of specific diseases (20, 21). Unfortunately, few studies have focused on the risk perceptions and responses of older adults or their caregivers. Lifshitz et al. (22) developed an instrument for assessing the risk perceptions of older adults. Kim et al. (23) assessed the relationship between caregiver information needs and their perceptions of fall risk. However, it is not clear what factors affect the risk perceptions and responses of informal caregivers.

The socio-ecological framework (SEF) can present a complex array of influencing factors in an intuitive and readily interpretable framework. The SEF considers that health and behavior are determined by factors ranging from the personal and interpersonal levels to the organizational, social, and political levels (24). This may advance health promotion programs from focusing on changes at the individual level to focusing on broader changes in the environmental context. Therefore, it has been increasingly adopted in research in health fields (25–27).

Informal caregivers are the gatekeepers to home care for older adults with disabilities. Understanding caregivers' risk perceptions may be a connecting point in promoting the safety of older adults at home. This study explored the impact of multiple levels of complex factors on the risk perceptions and responses of informal caregivers based on the SEF. The findings of this work are intended to provide a path for future work to promote the safety of home care for older adults with disabilities.

## Methods

### Study design

This study adopted a descriptive qualitative design (28). Semistructured interviews were conducted with 16 informal caregivers. The matter of risk perceptions of caregivers of older persons has rarely been studied, and a qualitative approach for this study was chosen to obtain an in-depth understanding.

### Setting and participants

The study was conducted in Zhenjiang, Jiangsu Province, China. Participants were informal caregivers of older adults with disabilities. Older adults' disabilities were determined using the Katz Index of Independence in Activities of Daily Living (29). The inclusion criteria of informal caregivers were as follows: 1) individuals aged over 18 years; 2) individuals who were relatives, partners, or friends providing principal assistance (nonpaid) to a home-dwelling disabled older adult. Caregivers with communication problems due to functional (deafness or aphasia) or intellectual impairment were excluded.

The first author contacted six public organizations that have connections with older adults with disabilities to recruit participants. With the help of the staff of these public organizations, the first author contacted the participants by telephone; the purpose and methods of the study were explained, and participant eligibility and interview times were confirmed. A purposive maximum variation sampling strategy was used to diversify participant characteristics (30). We considered sex, age, educational background, place of residence, years of care, and kinship with older adults to be relevant characteristics.

### Data collection

This study was performed from October 2020 to February 2021. The interview questions were developed based on the SEF and a review of the literature (31, 32), and then refined *via* discussions with three experts in geriatric nursing. An initial interview was performed with an eligible informal caregiver to assess the level of understanding and natural flow of the intended questions. Some questions were revised for clarity after several interviews.

The first author is a registered female nurse who is familiar with geriatric nursing and patient safety, has experience conducting qualitative studies and conducted all interviews. One-to-one interviews were performed at the participants' homes. Each interview started with broad questions to build rapport with participants. Following a brief exploration into how the caregiver defined risk, the interviews followed an interview guide (see Appendix 1). All participants underwent thorough interviews, and no participants declined to participate in or dropped out of this study. The interviewer had no previous relationship with the participant. No incentives were offered for participation. Interviews were audio-recorded with the participants' consent. The interviews lasted 26–52 min (*x* = 36 min). After the interview, field observations were conducted; we mainly observed the household environment (hygiene of the environment), the implementation of risk prevention measures (such as mattresses to prevent pressure ulcers and catheter fixation), and the quality of care (body cleaning, the presence of a pressure ulcer, etc.), and the findings were recorded.

### Data analysis

Data analysis was performed at the same time as data collection. NVIVO V.12 (QSR International) software was used to manage the data. The interviews were transcribed verbatim and analyzed using deductive content analysis (33).It involves preparation, organization, and reporting phases. During the preparation phase, the third and fourth authors were mainly responsible for transcription. The first and second authors reviewed each transcript and field notes several times in an immersive manner. During the organization phase, four levels of classification frameworks (individual level, familial level, community level, social level) regarding influencing factors were developed through discussion based on the SEF. Then, the first and second authors independently coded the transcript content line by line. Similar data were classified to form different topics and placed within one of the four levels, and any issues that arose over where data should be placed were discussed and agreed upon by both. Having placed the data within one of the four levels, it was then grouped into sub-themes by discussion. During the reporting phase, all findings were systematically presented. Caregivers contributing to each theme are shown in Appendix 2, and descriptive excerpts have also been identified. All participants were provided with a summary of the results at the conclusion of the study. Although code saturation was reached after 12 participants, an additional 4 caregivers were interviewed to achieve meaning saturation and develop a richly textured understanding of the issues (34).

### Rigor

The criteria recommended by Guba and Lincoln were used (35). Participants in our study had diverse ages, educational backgrounds, family economic statuses, and kinship backgrounds. Credibility was added through the combination of interviews and on-site observations of the safety care status of the older adults. All transcripts and researcher notes were analyzed by two researchers. The team had discussions regularly throughout the study. To achieve transferability, the maximum variation in sampling and sufficient descriptions of the phenomena were used to enhance the quality of the study. The consolidated criteria for reporting qualitative research guidelines were used to report the results (36).

### Ethical considerations

Ethics approval was obtained from the medical ethics committee (approval number: 2020-483). Written informed consent for sample collection, analysis and the publication of anonymised data was obtained from all participants. Each participant was interviewed in a private space to ensure their privacy, and all personal information obtained in this study was kept confidential. The study was compliant with the Declaration of Helsinki.

## Results

### Participant characteristics

Sixteen informal caregivers participated in the qualitative interviews. The mean age was 60.88 years (range, 36–78 years). Eleven caregivers lived in urban districts, and five lived in rural areas. Nine caregivers were female, and seven were male. The mean age of the older adults with disabilities was 76.06 years (range, 60–95 years). Their characteristics are shown in Table 1.

**Table 1:** Demographic characteristics of the informal caregivers and older adults with disabilities (*n* = 16 pairs).

**No**.	**Informal caregivers**						**Older adults with disabilities**		
	**Sex**	**Age**	**Residence**	**Education level**	**Years of care**	**Kinship with disabled elderly individual**	**Sex**	**Age**	**Degree of disability**
N1	Female	52	Urban	Undergraduate	8	Daughter	Male	76	Moderate
N2	Male	61	Urban	Undergraduate	2	Son	Female	86	Severe
N3	Male	36	Rural	Junior school	7	Son	Female	68	Moderate
N4	Female	78	Urban	Primary school	10	Spouse	Male	80	Severe
N5	Female	56	Urban	High school	5	Daughter-in-law	Female	91	Severe
N6	Female	62	Urban	Undergraduate	2	Daughter	Male	89	Severe
N7	Female	65	Rural	Illiteracy	3	Spouse	Male	68	Mild
N8	Male	72	Rural	Primary school	8	Spouse	Female	70	Mild
N9	Female	55	Urban	Junior school	5	Spouse	Male	61	Severe
N10	Female	63	Urban	Undergraduate	3	Daughter-in-law	Female	84	Severe
N11	Male	69	Rural	Primary school	20	Son	Female	95	Severe
N12	Male	63	Urban	Primary school	9	Relative	Male	79	Severe
N13	Female	55	Urban	Junior school	6	Spouse	Male	60	Severe
N14	Male	49	Urban	High school	4	Son	Female	72	Moderate
N15	Male	76	Rural	Primary school	5	Spouse	Female	73	Mild
N16	Female	62	Urban	Junior school	2	Spouse	Male	65	Moderate

### Identified themes

**Figure 1:**
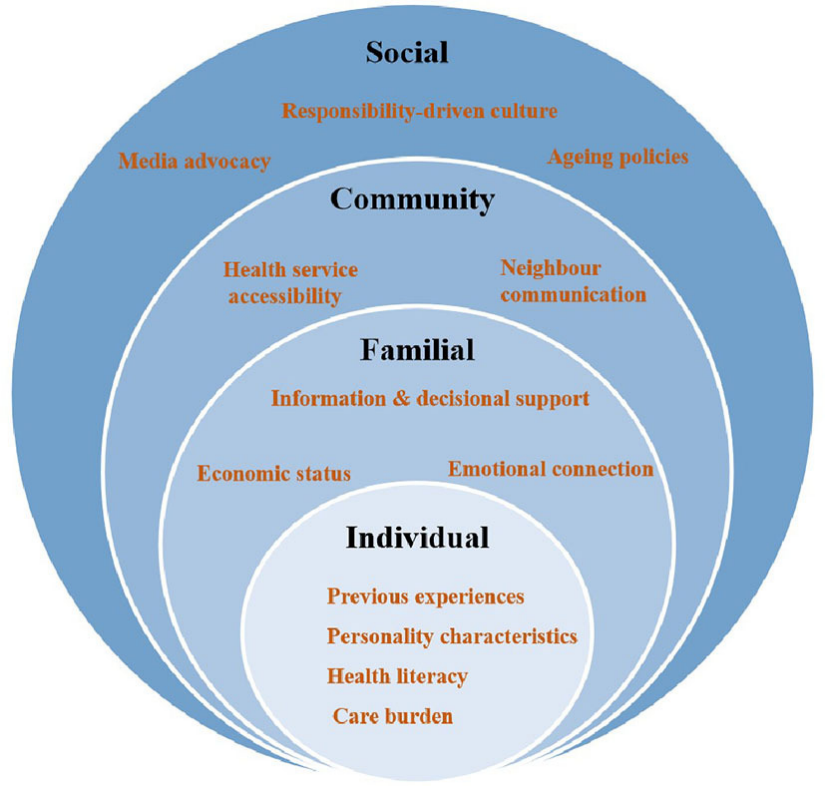
Socio-ecological framework showing the influences on risk perceptions and responses of informal caregivers.

The SEF contains many variables at five levels. In specific research, some scholars choose to adjust the model according to the specific research population and research content (25, 37). Here, we present the influences on risk perceptions and responses that emerged as sub-themes within four levels of the SEF, as presented in Figure 1.

### Individual level

#### Sub-theme 1: Previous experiences

Caregivers often drew strength from their care practices and experiences. Similar experiences gradually solidified into habits, impacting the caregivers' perceptions and responses to risk. Nursing experience was usually closely related to the length of care and the occurrence of adverse events. Falls were the most common adverse event among older adults in the household. Falls were also the most common risk perceived by the caregivers.

“*In the first year after returning from the hospital, my husband's nasogastric tube was often pulled out, which was very troublesome. Later, I was very careful.” (N9)*

Past events without serious consequences could also weaken the caregivers' perceptions of severity.

“*My mother falls several times a day. She is small and light. She has fallen so many times without any harm.“ (N3)*

#### Sub-theme 2: Personality characteristics

Personality characteristics were related to risk perception. Some overly optimistic caregivers tended to underestimate the probability of risks. Weinstein refers to this underestimation as “optimistic bias” (38). They believed they were better at nursing than other caregivers and that the older adults they cared for were less likely to experience undesirable adverse events than other older adults.

“*I have taken care of the older adult for several years. You can see that I have taken care of them very well. I believe that the older adult whom I take care of will not be at any risk.” (N12)*

Some caregivers tend to overestimate the probability of risks. They are afraid of risks and restrict the behaviors of older adults to avoid risks.

“*My wife often fell when walking; I always worry about accidents. Now I let her stay at home and walk less.” (N8)*

#### Sub-theme 3: Health literacy

Health literacy is “the degree to which individuals have the capacity to obtain, process, and understand basic health information and the services needed to make appropriate health decisions” (39). Caregivers who were young and had a higher level of education seemed to have better health literacy. Therefore, they were more likely to obtain risk information.

“*Before my mother was discharged from the hospital, I had already consulted the nurse by asking what I should pay attention to at home.” (N5)*

“*I'm not educated, and I cannot read books and newspapers, and I do not truly understand.” (N7)*

#### Sub-theme 4: Care burden

Elderly individuals with disabilities, especially those with dementia, are in great need of care. The physical and mental health of caregivers are affected, and the perceived burden of caregivers weakens their risk perceptions and responses.

A 78-year-old woman who takes care of her 80-year-old husband said, “*I am not in good health, and I am exhausted. I feel dizzy every day, and I am truly powerless to care about these.”* She was the only caregiver from Monday to Friday. Their house was messy and smelled of urine. *(N4)*

Some caregivers with a high care burden expressed a resignation to fate.

“*I have to take care of my mother, my son is working outside, and my grandson also needs our care. The elderly man is so old, and some things will inevitably come. I do not want to struggle; let nature take its course.” (N11)*

### Familial level

#### Sub-theme 5: Economic status

The costs of responding to risk hinders individuals from adopting healthy behaviors. There were many factors that impacted the caregivers' use of risk prevention behaviors. A family's ability to afford such protections was the main factor. In families with good financial status, the caregivers generally paid more attention to health and safety, and they were more actively engaged in the transformation of the family environment and the purchase of care products. N9 and N13 mentioned that they bought anti-pressure ulcer air cushions and walking aids for the older adults.

In families with poor financial status, the caregivers often needed to consider the economic costs and possible benefits of risk intervention.

N15 mentioned that his wife had fallen many times. We observed that there were many steps in his home, and the toilet floor was not nonslip. N15 said, ”*It will cost a lot of money to redecorate the house, and my retirement salary is low.”*

In addition, older adults with good economic status who provided economic support to their children received greater attention regarding safety.

N5, the unemployed daughter-in-law of an older adult, said, “*My mother-in-law has a very high pension. My job is to take good care of her and ensure her safety.”*

#### Sub-theme 6: Emotional connection

Emotional connection is manifested in two aspects: One is the relationship between the caregiver and the elderly individual, and the other is the emotional support provided by family members for the caregiver. Four caregivers mentioned that the older adults used to be so kind to them, and they must take good care of her or him.

There is a Chinese proverb that says there is “no dutiful son before the sick bed.” Providing care for older adults with disabilities for a long period of time weakened family intimacy and the caregivers' concerns about safety. When there was no adequate family and social support, this threat of neglect became more prominent.

N11, the son of a disabled woman residing in the countryside, said, “*My mother has been paralyzed and bedridden for twenty years. I have two brothers, but they do not care. She is now in her nineties and has no quality of life. The consequences of the risks no longer matter….”* The elderly woman was found to suffer from a large pressure sore, and the room was noted to smell foul.

Family expectations were also an emotional factor that affected the caregivers' risk perceptions.

“*My father lived with me. I promised my brother I would take good care of him, so I was very nervous if my father had any problems.” (N6)*

#### Sub-theme 7: Informational and decisional support

Informational support from family members improved the risk perceptions of older adults and their caregivers.

N14, a son living with his parents, said, “*Whenever I read articles, either online or in the newspaper, that are relevant to adverse events at home, I tell my parents to be careful*.”

Elderly individuals in China are generally economical, and they are often unwilling to spend extra money on their personal safety or health. The involvement of their offspring in making decisions changes their safety views.

N1 said, “*The bathroom is dark and slippery; they think it is very expensive to remodel. I insisted on bathroom modification.”*

### Community level

#### Sub-theme 8: Health services accessibility

Health services can help informal caregivers identify risks and guide risk prevention, but there are few opportunities to receive these services. Three caregivers mentioned that family ward services were provided for older adults with disabilities, but these services mainly provided medical diagnoses and treatment. Minimal risk assessment and guidance were available.

“*Doctors and nurses come here every month. They come to take blood pressure, do a cardiac auscultation, etc. They don't tell us what risks we should pay attention to.“ (N7)*

Transitions are particularly problematic when patients leave the hospital to return to their home. In China, hospital services and community care services are not yet linked in most cities.

“*When my husband had just been discharged from the hospital, no one told me what to do. Accidental incidents occurred several times.” (N9)*

#### Sub-theme 9: Neighbor communication

Neighbor communication is the main social communication style for older adults in China. The risk events experienced by surrounding neighbors also contributed to the personal experiences of the participants and, to a certain extent, influenced their judgment of the possibility of risks and the way they reacted to them.

“*I heard from others that an old man died at home after a fall, so I paid special attention not to let the older adult fall.” (N16)*

Some caregivers were influenced by what was happening to those around them and expressed fear of adverse events.

“*An aunt went to the toilet and suddenly died. The doctor said that it might have been a pulmonary embolism; I am worried that this will happen to my mother.“ (N10)*

### Social level

#### Sub-theme 10: Responsibility-driven culture

The informal caregivers' risk perceptions and responses to home care safety for older adults was affected by ethical factors. Six caregivers mentioned that it was their responsibility to take good care of the older adults.

“*An old man in our village lived alone at home. He fell to his death in the toilet at night. Everyone said that it was not a filial duty*.” *(N3)*

#### Sub-theme 11: Media advocacy

People often learn about risk issues through various information transmission channels, such as magazines, the radio, television, newspapers, and the internet. However, it is difficult for home caregivers who are older and less educated to gain information.

Six caregivers mentioned the lack of information channels and single content provided by the media. The existence of the digital divide also makes it difficult for caregivers to obtain information from new types of media channels. Caregivers with a higher level of education seemed to have more channels for obtaining information, but information on the internet was scattered and of uneven quality. It was difficult for caregivers to judge whether information was correct or not.

“*I do not know if the information online is credible or not. I still hope to get professional guidance from a doctor.” (N2)*

#### Sub-theme 12: Aging policies

The policy level relates to the various regulations, laws and initiatives introduced by governing bodies at the local and national levels. Two caregivers mentioned that the government provided home modifications. Three elderly people mentioned that they enjoyed the government's family bed ward medical insurance for older adults with disabilities.

“*I heard that in some districts, handrails in the toilets were installed by the government for elderly individuals. It is true that elderly individuals are prone to fall in the toilets.” (N1)*

## Discussion

This was the first qualitative study to explore informal caregivers' risk perceptions and responses to the home care safety of older adults with disabilities. The SEF allowed us to include various factors that affect caregivers' risk perceptions and responses into a framework for comprehensive consideration. This stratification enabled a deeper perspective to understand the safety of home care for older adults with disabilities, which is lacking in previous studies.

The findings of the present study revealed that individual factors affecting risk perceptions and responses include the following: First, caregivers generally rely on accumulated experiences for risk awareness. However, the accumulation of experience may take time, and this aggregation may come at a “high price” for the health of older adults and their caregivers. Many studies have indicated that training and sharing experiences with others can help to effectively overcome these challenges (40, 41). Second, personality characteristics can lead to cognitive biases, which was confirmed in a systematic review (42). Optimists may have distorted predictions of future risk events. A study showed that optimism increased with experience, and it appears that optimism arises because people persistently overestimate the degree of control that they have over an event (43). Protection motivation theory (PMT) states that threat appraisal is a cognitive mediating process, and the perception of risk severity and vulnerability is a promoter of intention and behavior (44). Because of the insufficiency of information resources, few caregivers had a clear and comprehensive understanding of the risks of in-home care for older adults, so it was difficult to accurately assess the severity and vulnerability of these risks. Third, health literacy impacts risk perceptions and responses. A number of studies have found a positive relationship between health literacy and health outcomes (45, 46), and there are also initiatives to promote patient safety through health literacy (47). Fourth, care burden cannot be ignored; most of the caregivers in China were the sole full-time caregivers and had little time for themselves (48). The collision of the intergenerational family life cycle increases the plight of caregivers. Depression, anxiety, and sleep problems are the main challenges for caregivers, and these are all possible impact factors influencing their risk perceptions and coping.

It was found that an adequate family environment has an active role in establishing proper risk perceptions and responses of caregivers. Older adults with disabilities were found to face higher levels of health expenditures. The impact of family financial status on risk perception may be related to better economic conditions that increase the affordability of additional expenditures caused by disability. In addition, the economic status of elderly individuals also determines whether they can receive more attention regarding their safety. For families with poor financial status, caregivers appeared to attend more to response costs and benefits. Emotional influence and human feelings are important influencing factors for understanding risk perception. The relationship between the caregiver and the elderly individual determines the caregiver's attention to the safety of the elderly individual. Disability is an important stressor that affects the mental health of elderly individuals, and many caregivers have also reported high levels of stress, fatigue, the development of depression, and other health problems (49). Emotional support makes caregivers feel that they are being cared for and respected, which serves to decrease the burden and resolve mental health issues; therefore, it also affects the caregiver's risk perceptions and responses. Informational and decisional support from offspring promotes the risk perceptions and coping of caregivers. Younger children have greater contact with the outside world and encounter more information regarding safety issues. The successful communication of such information to caregivers improves their risk perception and coping skills. In addition, family caregivers often face difficulties in decision-making or an unclear willingness for decision-making. Concern and support from offspring may aid in making safety decisions.

At the community level, the impact of community health services on the risk perceptions and coping of caregivers was not fully demonstrated in this study. This may be related to the fact that China has only recently begun to offer home health services, and risk-management standards and procedures have not yet been established. Informal communication between neighbors plays a role in promoting the risk perceptions of caregivers. Neighbor communication disseminates information quickly and has a strong influence, but it is easy for this information to be exaggerated or misunderstood. Professionals are required to guide caregivers in using this communication method effectively.

At the social level, the Chinese are more deeply influenced by the traditional culture of filial piety (50). We found that the culture of filial piety affects the risk perceptions and responses of caregivers. The sense of responsibility is not only a belief but also a moral drive. Media advocacy has emerged as an important communication-based public health intervention, and it also affects the risk perceptions of caregivers. According to PMT, the risk perception process is initiated by the information source (51). In our study, caregivers who were older and less educated reported personal barriers to information acquisition. Some caregivers noted the lack of effective channels for obtaining information. Previous works have found that the number of information acquisition channels had a statistically positive impact on risk perceptions at the levels of 1 and 0.1% (32). This suggests that a variety of means should be used to enable more caregivers and the public to obtain accurate information. It has also been shown that perceived risk emanates from the degree of trustworthiness of the provided information (52). For uncertain health risks, caregivers may trust information from health care professionals.

Taken together, we suggest that comprehensive, realistic, and achievable strategies are needed to improve the risk perceptions of informal caregivers. First, strategies should be adopted to empower caregivers to positively participate in risk prevention. Caregiver training is essential, and some studies have shown that providing personalized feedback about risks leads participants to improve their risk perceptions (53, 54). The Chinese government needs to develop a sound long-term care insurance system to provide safe care support for older adults with disabilities, and the health administrative department should develop regulations and establish supervisory groups to oversee the implementation of these services regularly. Second, familial support should be encouraged. Recently, the Chinese government has encouraged elderly individuals and their children to live near each other, which aims to promote emotional connections and intergenerational support. Third, the media should therefore more closely collaborate with healthcare professionals, and information should be disseminated not only *via* formal channels but also through informal channels. Information should be disseminated in a more “user-friendly” format to narrow the digital divide for caregivers. Fourth, policymakers should implement caregiver support systems for informal caregivers to lessen the caregivers' economic and family burdens. Policies to support families also deserve more attention.

## Strengths and limitations

However, there are some limitations that require consideration. First, this was a small qualitative study of informal caregivers in China, and the study was performed in only one city. It is therefore subject to bias because of regional selection and research conditions. Second, we only performed a single interview with each caregiver, so we could not gain an in-depth understanding of the topic. Future studies involving more regions, more tracking, and more participants are needed to validate the findings of this report. Moreover, quantitative research is needed to assess the interconnections between different elements.

## Conclusions

The risk perceptions and coping behaviors of informal caregivers are affected by many factors. We found that the SEF proved to be an effective means of identifying and presenting these underpinning influences. We believe that our findings could facilitate a better understanding of the determinants that underlie the risk perceptions and responses of informal caregivers. It also provides a basis for improving caregivers' risk perceptions and home care safety. Comprehensive strategies are needed in future intervention studies to improve the risk perceptions of informal caregivers providing in-home care.

## Data availability statement

The original contributions presented in the study are included in the article/Supplementary material, further inquiries can be directed to the corresponding author.

## Ethics statement

The studies involving human participants were reviewed and approved by Research Ethics Board of the First Affiliated Hospital of Chongqing Medical University. The patients/participants provided their written informed consent to participate in this study.

## Author contributions

SC: conceptualization, methodology, interviewing, formal analysis, and writing–original draft. HH: formal analysis and writing–original draft. SB and MF: data curation. YLia and YLiu: assisting in data collection. QZ: conceptualization, funding acquisition, supervision, and writing–review and editing. All authors contributed to the article and approved the submitted version.

## Funding

This study was funded by Medical Scientific Research Project of Chongqing (Award Number: 2021MSXM054).

## Conflict of interest

The authors declare that the research was conducted in the absence of any commercial or financial relationships that could be construed as a potential conflict of interest.

## Publisher's note

All claims expressed in this article are solely those of the authors and do not necessarily represent those of their affiliated organizations, or those of the publisher, the editors and the reviewers. Any product that may be evaluated in this article, or claim that may be made by its manufacturer, is not guaranteed or endorsed by the publisher.
