# Supplementary material for: What influences informal caregivers' risk perceptions and responses to home care safety of older adults with disabilities: A qualitative study
Source: Front Public Health. 2022 Aug 24;10:901457. doi: 10.3389/fpubh.2022.901457 (PMC9449117; doi:10.3389/fpubh.2022.901457)
Supplement: Supplementary file 1 [file Table_1.DOC]

Appendix 1

**Interview outline**

| 1. Tell me a little about your experience caring for the older people. 2. What do you know about the potential risks (unintended harm) that may occur to the older people in home care? 3. How do you know these risks? 4. How do you view the undesirable consequences of these risks, and how likely do you think these risks may occur to the elderly you care for? 5. Can you describe what measures your practice employs to ensure care safety? 6. What difficulties do you have in taking risk prevention measures or behaviors? 7. What support do you acquire to help you better implement safe home care for the older people? 8. What do you think are your personal factors that may affect risk perception and behavior in a practice? 9. What do you think are familial factors that may affect risk perception and behavior in a practice? 10. What do you think are community or social factors that may affect risk perception and behavior in a practice? |
| --- |
