# Supplementary material for: What influences informal caregivers' risk perceptions and responses to home care safety of older adults with disabilities: A qualitative study
Source: Front Public Health. 2022 Aug 24;10:901457. doi: 10.3389/fpubh.2022.901457 (PMC9449117; doi:10.3389/fpubh.2022.901457)
Supplement: Supplementary file 2 [file Table_2.DOC]

Appendix 2

Caregivers contributing to each theme

| Number | **Individual level** | | | | **Familial level** | | | **Community level** | | **Social level** | | |
| --- | --- | --- | --- | --- | --- | --- | --- | --- | --- | --- | --- | --- |
|  | Previous experience | Personality characteristics | Health literacy | Care burden | Economic status | Emotional connection | Informational and decisional support | Health services accessibility | Neighbour communication | Responsibility-driven culture | Media advocacy | Ageing policies |
| N1 | ✓ |  | ✓ | ✓ |  | ✓ | ✓ | ✓ |  |  |  | ✓ |
| N2 | ✓ |  | ✓ |  |  | ✓ | ✓ |  |  | ✓ | ✓ | ✓ |
| N3 | ✓ |  |  | ✓ | ✓ |  |  | ✓ |  | ✓ |  |  |
| N4 | ✓ |  |  | ✓ | ✓ |  |  |  |  |  | ✓ |  |
| N5 |  |  | ✓ |  | ✓ |  |  | ✓ |  |  |  | ✓ |
| N6 | ✓ |  | ✓ | ✓ | ✓ | ✓ | ✓ | ✓ |  |  |  | ✓ |
| N7 | ✓ |  | ✓ | ✓ |  |  |  | ✓ | ✓ |  | ✓ |  |
| N8 | ✓ | ✓ |  | ✓ |  |  |  |  |  | ✓ | ✓ |  |
| N9 | ✓ |  |  | ✓ | ✓ | ✓ | ✓ | ✓ | ✓ |  |  |  |
| N10 | ✓ |  | ✓ |  |  |  |  |  | ✓ | ✓ |  | ✓ |
| N11 | ✓ |  |  | ✓ |  | ✓ |  | ✓ |  |  |  |  |
| N12 | ✓ | ✓ | ✓ |  |  |  |  |  |  | ✓ | ✓ | ✓ |
| N13 | ✓ |  |  | ✓ | ✓ | ✓ |  | ✓ |  | ✓ | ✓ |  |
| N14 |  |  |  |  |  |  | ✓ |  |  | ✓ |  |  |
| N15 | ✓ |  |  | ✓ | ✓ |  |  |  | ✓ |  | ✓ |  |
| N16 |  | ✓ | ✓ |  |  |  |  |  | ✓ |  |  |  |
| Number of caregivers contributing to each theme | 13 | 3 | 8 | 10 | 7 | 6 | 5 | 8 | 5 | 7 | 7 | 6 |
